# Small Airways Disease as a Novel Target for Mepolizumab in Asthma—The SASAM Prospective Real-Life Study

**DOI:** 10.3390/jcm14092928

**Published:** 2025-04-24

**Authors:** Matteo Bonini, Cristina Boccabella, Francesca Cefaloni, Eugenio De Corso, Federico Donfrancesco, Enrico Schiavi, Luca Richeldi

**Affiliations:** 1 Department of Public Health and Infectious Diseases, Sapienza University of Rome, 00185 Rome, Italy; matteo.bonini@uniroma1.it; 2 National Heart and Lung Institute (NHLI), Imperial College London, London SW7 2AZ, UK; 3 Department of Cardiovascular and Pulmonary Sciences, Università Cattolica del Sacro Cuore, 00168 Rome, Italy; cristina.boccabella@gmail.com (C.B.);; 4 Otorhinolaryngology Unit, Fondazione Policlinico Universitario Agostino Gemelli IRCCS, 00168 Rome, Italy; 5 Unità Operativa Complessa di Pneumologia, Fondazione Policlinico Universitario A. Gemelli IRCCS, 00168 Rome, Italy

**Keywords:** asthma, impulse oscillometry, mepolizumab, small airways, small airway disease

## Abstract

Mepolizumab represents an effective strategy for severe eosinophilic asthma. Small airways disease (SAD) defines a peculiar asthma phenotype related to worse disease control. Limited and indirect findings are currently available on the effect of mepolizumab on SAD. **Objectives:** We investigated the impact of mepolizumab on SAD assessed through impulse oscillometry (IOS) and spirometry. As secondary outcomes, we tested the correlation between SAD and clinical, functional and inflammatory parameters. **Methods:** This is a prospective cohort study including severe eosinophilic asthmatics eligible for mepolizumab performed between 2021 and 2023. IOS (R5–R20) and spirometry (FEF25-75%, TLC%, RV/TLC%) parameters were assessed at baseline and over 1 year of mepolizumab. Other functional (FEV1%), clinical (ACT, number of asthma exacerbations/previous year, use of OCS) and inflammatory data (BEC and FeNO) were concomitantly collected for correlations. **Results**: A total of 18 patients (mean age 61.1 ± 12.0 y; 10 (55.5%) female) were included. Longitudinal data from 16 patients showed that R5–R20 significantly improved after 12-months treatment (*p*: 0.03), as well as FEF25-75% (*p*: 0.04) and TLC% (0.04). FEV1% and ACT showed a concomitant improvement (*p*: 0.03 and <0.01, respectively). All the steroid-dependent subjects discontinued OCS after 3 months and the percentage of subjects experiencing exacerbations significantly decreased (*p*: <0.01). As per drug effect, BEC consistently decreased (*p*: <0.01). The decrease in R5–R20 correlated with an improvement in FEF25-75% (r: −0.40 *p*: 0.048) and ACT at T12 (r: −0.59 *p*: 0.02). **Conclusions**: Twelve months treatment with mepolizumab improved R5–R20, suggesting its additional role as a targeted treatment for distal lung regions. This improvement also correlated with a clinically relevant amelioration of asthma symptoms.

## 1. Introduction

Despite the current availability of a broad spectrum of effective medications for the majority of asthmatics, many of them remain uncontrolled and severe, causing a substantial health burden and resource expenditure. In fact, although patients with severe asthma account for 5–10% of asthmatics, their care is estimated to cause more than 60% of the costs associated with the disease, including medications, days of hospitalization, admissions to the emergency room and unscheduled consultations. Severe asthma is a heterogeneous syndrome accounting for several phenotypes and represents a complex disease requiring an expert and multidisciplinary approach, as well as the use of multiple and tailored treatment strategies. Although pharmacological options were previously limited, the emergence of biologics has provided promising targeted therapy for these patients. Biologics target specific inflammatory pathways involved in the pathogenesis of asthma, particularly in patients with a type 2 (T2) inflammation-driven endotype. However, much remains unclear regarding the best approach to manage these patients, as well as the pathophysiological mechanisms underlying the disease [1,2,3,4].

Historically, asthma assessment mainly involved the functional evaluation of the large airways through spirometry, considering bronchial obstruction as the landmark for the diagnosis and severity staging. However, it is now widely accepted that small airways (SAs), those with an internal diameter < 2 mm, are deeply involved in the pathogenesis of asthma and represent the major determinant of airflow obstruction [5,6,7]. From a clinical perspective, small airways disease (SAD) has been associated with negative asthma outcomes, such as wheezing, night-time awakenings and exacerbations. Interestingly, several studies have shown that SA abnormalities, even in patients with normal spirometry, are associated with a lack of asthma control [8,9]. Noninvasive techniques including body plethysmography, single and multiple breath nitrogen washout, impulse-oscillometry (IOS), fraction exhaled NO at multi-flow, sputum induction and high-resolution chest CT (HRCT) have been used to assess the extent of SAD in asthma [7,10,11]. IOS, using pressure waves at frequencies from 5 to 35 Hz, can directly evaluate SA resistance during a tidal breathing maneuver resulting in being easier to perform and more sensitive in detecting SAD when compared to other direct and indirect functional tests. A recent multicenter study (ATLANTIS) aimed at estimating SAD in asthma, has developed a score, based on functional, clinical and radiological variables, to predict the extent of SA involvement in patients with asthma and its correlation with clinical features and prognosis. Importantly, IOS assessment, through R5–R20 (resistance at 5 Hz and 20 Hz) reflecting small- to mid-sized airways resistance, was observed to be the best predictive marker of uncontrolled asthma. According to the literature findings, R5–R20 baseline values > 0.07 kPa/L/s support the evidence of SAD [12]. Furthermore, a significantly increased SAD score was found in patients with severe asthma (GINA step 5), frequent exacerbations, multiple comorbidities, poor symptom control and eosinophilic inflammation [13]. Nevertheless, these measures are not usually part of the evaluation of asthmatic patients and of the monitoring of the drug effectiveness for severe asthma [14,15].

Considering the above cited evidence, we believe that IOS may be the most appropriate diagnostic tool to directly measure SA involvement and monitor biologic effects at the lung periphery.

Mepolizumab represents an effective biologic strategy for the treatment of severe eosinophilic asthma, determining symptom improvement, exacerbation rate reduction and steroid-sparing effect [16,17,18,19].

However, limited findings are available about the effect of mepolizumab on SAD, when directly assessed through impulse oscillometry, particularly through the R5–R20 parameter, which may offer valuable insights. Whether an improvement in R5–R20 values is associated with better clinical outcomes during mepolizumab treatment remains unknown. In the context of asthma phenotyping and personalized therapy for patients with severe eosinophilic asthma, this analysis could reveal a novel therapeutic target for mepolizumab.

On that premise, we hypothesize that in severe asthmatics, mepolizumab might exert an improvement in SAD and that the evaluation of SA before starting and during biologic treatment may represent a distinctive marker of response and a novel target for a preferential use of this drug in comparison to other available biologics for severe eosinophilic asthmatic patients.

## 2. Materials and Methods

### 2.1. Study Design and Population

This was a 12-month, single-site observational prospective cohort study including consecutive adult subjects (≥18 years) diagnosed with severe asthma (according to the ATS/ERS guidelines and GINA recommendations) [4,19] and referred to the Asthma Centre of the Fondazione Policlinico Universitario A. Gemelli—IRCCS. After a multidisciplinary team (MDT) discussion, in view of the lack of asthma control, despite optimized inhalation therapy, together with a documented peripheral blood eosinophil count > 150/μL at baseline or ≥300 cells/µL historically, subjects were considered eligible for a step-up treatment with mepolizumab. In order to be included in this study, patients needed to be able to sign an informed consent and perform reproducible IOS at baseline.

As an observational prospective cohort study, this design carries certain limitations, including the potential for selection bias and data missing. To minimize selection bias, we enrolled consecutive patients meeting the guideline-based criteria described above, thus ensuring consistency in the baseline characteristics. Information bias was reduced by relying on objective, reproducible measurements performed using standardized protocols and validated equipment described below. Furthermore, by prospectively collecting data at defined time-points we aimed to enhance data reliability and reduce follow-up biases.

All the included subjects received mepolizumab 100 mg subcutaneously every 4 weeks. Demographic data (i.e., age, gender, body mass index-BMI, smoking habits, atopic status) were collected at baseline from clinical records. SA measurements assessed through IOS (R5–R20) and global spirometry (forced expiratory flow between 25% and 75% of vital capacity–FEF25-75% predicted, total lung capacity–TLC % predicted; residual volume/TLC-RV/TLC %) were evaluated at baseline and at 3 (T3), 6 (T6) and 12 (T12) months of mepolizumab treatment as per clinical practice. Forced expiratory volume in the 1st second (FEV1) % predicted, forced expiratory volume in the 1st second/forced vital capacity (FEV1/FVC), Asthma Control Test (ACT), number of asthma exacerbations occurred in the previous year, OCS use, blood eosinophilic count and fractional exhaled nitric oxide (FeNO) were also measured for functional, clinical and laboratory trends and correlations at each time-point.

The main procedures are described below:

- Pulmonary function tests and body plethysmography were performed using the Q-Box (COSMED—The Metabolic Company, Rome, Italy) [20];
- IOS: According to the literature evidence, baseline values > 0.07 support the evidence of small airways dysfunction [10,21]. All the procedures were performed with a Vyaire IOS device (Vyaire Medical, Mettawa, IL, USA);
- Airway inflammatory markers (FeNO); FeNO measurements were performed in accordance with ATS/ERS recommendations at a flow rate of 50 mL/s [22], using a chemiluminescence analyzer (NIOX Flex, Aerocrine AB, Solna, Sweden).

This study was approved by the Local Ethical Committee of the Fondazione Policlinico Universitario A. Gemelli-IRCCS (protocol ID 3999) and was registered on ClinicalTrials.gov (NCT05040997). This study was performed between 2021 and 2023. Written informed consent was obtained from each patient for the use of personal data.

### 2.2. Study Objectives

The primary study outcome was the evaluation of mepolizumab effects on SAD through direct and indirect functional parameters. The considered primary endpoint was the R5–R20 value and its changes from baseline over a 1-year follow-up period—at 3 (T3), 6 (T6) and 12 (T12) months from the first mepolizumab dose. Along with the IOS assessment, we also evaluated indirect SAD parameters such as FEF25-75% pred, TLC% pred and RV/TLC% at each time point.

As secondary outcomes, we assessed in parallel the changes in functional (FEV1% pred.; FEV1/FVC%), clinical (ACT, percentage of asthma exacerbations occurred, OCS treatment use) and inflammatory parameters (blood eosinophilic count and FeNO) from baseline over a year of treatment.

### 2.3. Statistical Analysis

This was a pilot proof of concept prospective observational study and, therefore, did not imply a preliminary definition of the minimal sample size. Statistical analyses were performed with GraphPad Prism 10. Data were tested for normality using the D’Agostino–Pearson test and accordingly expressed as mean (±Standard Deviation—SD) or median (Interquartile Range—IQR). Categorical variables were considered as number of cases and percentages (N and %). For normally distributed continuous variables comparisons from T0 to T3, 6, and 12 were performed using the T test and the Wilcoxon matched-pairs signed rank test for non-parametric data. Categorical data were compared with Chi-square test. Correlations were performed using the Pearson r test. A *p*-value < 0.05 was considered statistically significant. Patients with non-reproducible IOS measurements at baseline (primary outcome) and lost to follow-up were excluded for the longitudinal analysis.

## 3. Results

Twenty consecutive patients entered this study. IOS maneuvers performed at baseline did not fulfil standards of quality in two patients, who were therefore excluded from the final data analyses. Baseline characteristics of the 18 enrolled patients are summarized in Table 1. Briefly, mean (±SD) age and BMI were 61.1 ± 12.0 years and 27.1 ± 5.10 kg/m^2^, respectively. Ten subjects (55.5%) were female, five subjects (27.7%) were either former or current smokers. Atopy (sensitization to at least one perennial or seasonal aeroallergen detected by skin prick tests or radioallergosorbent test—RAST) was recorded in 61.1% of the population. The entire sample was receiving background inhaled therapy with a medium-to-high dose of ICS/LABA; 17 out of 18 (94%) patients were also treated with LAMAs. No change in the inhaled treatment occurred over the study period.

At baseline, 15 subjects (83.3%) out of 18 were found to have SAD, defined as R5–R20 > 0.07 kPa/L/s, the mean (SD) R5–R20 for the whole population was 0.25 ± 0.15 kPa/L/s. Mean FEV1 was 1.70 ± 0.78 L, FEV1% pred. 68.0 ± 24.1% with a mean Tiffeneau Index (FEV1/FVC%) of 59 ± 21.6%. Mean (SD) FEF25-75% pred. was 31.9 ± 23.0%, TLC% pred. was 96.4 ± 16.2% and RV/TLC% was 45.0 ± 10.7%. In regard to disease control, the mean (SD) ACT score was 14.7 (±5.6), while 15 patients (83.3%) reported at least one exacerbation requiring OCS in the previous year and 4 patients (22%) underwent maintenance OCS treatment (mOCS). Blood eosinophil count (BEC) median (IQR) was 261 (220–520) cell/mm^3^ and mean (SD) FeNO was 47.8 (±36.5) ppb.

Analysis of the primary endpoint, performed in a sample of 16 patients after having excluded 2 lost to follow-up subjects, showed a change in R5–R20 value over 1 year treatment period from a mean (SD) value of 0.24 (±0.15) kPa/L/s at baseline to 0.25 (±0.21) kPa/L/s at T3 (*p*: 0.45), 0.19 (±0.18) kPa/L/s at T6 (*p*: 0.05) and 0.18 (±0.18) kPa/L/s atT12 (*p*: 0.03). The absolute number of subjects with SAD (i.e., R5–R20 > 0.07 kPa/L/s) reduced from 13 (81%) at baseline (10 at T3 and T6, *p*: 0.43) to 9 (56%) at T12, despite not significantly (*p*: 0.13).

**Figure 1:**
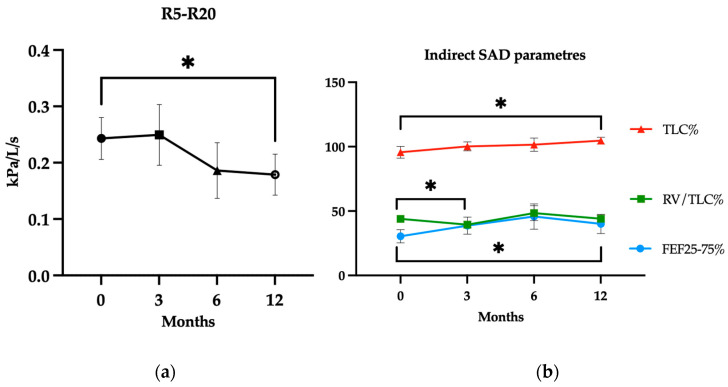
(**a**) Mepolizumab effectiveness on R5–R20 (IOS assessments of SAD. (**b**) Mepolizumab effectiveness on spirometry measurements: FEF25-75% predicted, TLC% predicted and RV/TLC% changes over 12 months. Data are expressed as mean ± SEM. *: *p*-value < 0.05. For clarity, only statistically significant differences have been reported.

The improvement of SAD was substantially confirmed by indirect parameters, such as FEF25-75% pred., starting from a mean (SD) of 30.4 ± 20.6% at baseline (38.7 ± 25.6%, *p*: 0.09 at T3; 47.5 ± 34.1%, *p*: 0.06 at T6) to 41.4 ± 28.8 at T12 (*p*: 0.04) and TLC% pred. increasing from the mean (SD) value of 95.6 ± 17.1% at baseline (100.2 ±12.3%, *p*: 0.21 at T3; 101.5 ± 17.1%, *p*: 0.26 at T6) to 104.7 ± 9.3% (*p*: 0.04) at T12 (Figure 1). The mean (SD) of RV/TLC% was 43.9 ± 9% at baseline (39.48 ± 10.1%, *p*: 0.01 at T3; 48.4 ± 19.0%, *p*: 0.26 at T6) and 44.9 ± 9.3% (*p*: 0.12) after 12 months.

Mean (SD) FEV1% pred. changed from a baseline value of 71.4 ± 22.9% (82.3 ± 34.3%, *p*: 0.05 at T3, 83.1 ± 33.4%, *p*: 0.06 at T6) and 80.8 ± 26.7% after one year of treatment (*p*: 0.03).

Clinical parameters showed a significant improvement in ACT score, namely from 15.1 ± 5.8 at baseline (19.0 ± 4.6, *p*: <0.01 at T3; 19.6 ± 3.5, *p*: <0.01 at T6) reaching the value of 20.3 ± 4.4 at T12 (*p*: <0.01). The four (25%) subjects with OCS discontinued OCS treatment after 3 months of mepolizumab, (0 at T3 and T6; *p*: 0.10) maintaining their suspension until T12 (*p*: 0.10). The N(%) of subjects experiencing at least one exacerbation in the previous year decreased from 15 (91) to 1 (6.2), *p*: <0.01 at T3 and 2 (12.5) *p*: <0.01 at T6, with a total amount of 3 (18.7) in the 12 months, *p*: <0.01. Blood eosinophil count values changed from a median (IQR) of 250 (220–477) cell/mm^3^ at baseline (70 (40–130) cell/mm^3^, *p*: <0.01 at T3; from 40 (20–111) cell/mm^3^ at T6, *p*: <0.01) to 50 (40–90) cell/mm^3^ (*p*: <0.01) after 12 months therapy. The mean (SD) FeNO value (ppb) was 49.2 ± 39.4 at baseline (44.5 ± 43.6 at T3, *p*: 0.35; after 6 months was 52.8 ± 48.5, *p*: 0.24) and 43.1 ± 26.4 at T12 (*p*: 0.07). All the presented results are reported in Table 2.

**Figure 2:**
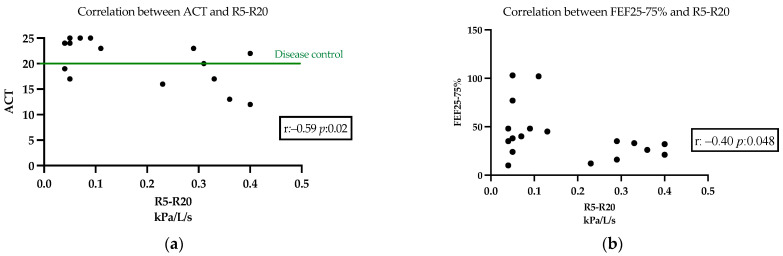
(**a**) Pearson r correlation between ACT and R5–R20 after 12 months of mepolizumab treatment (r: −0.59 *p*: 0.02). (**b**) Pearson r correlation between FEF25-75% predicted and R5-R20 after 12 months of mepolizumab treatment (r: −0.40 *p*: 0.048).

We found a significant correlation between R5–R20 and ACT values (Pearson r: −0.59 *p*: 0.02) measured after 12-months therapy with mepolizumab. In addition, a weak correlation was found between R5–R20 and FEF25-75% (Pearson r: −0.40 *p*: 0.048) at the same time point. The results are reported in Figure 2.

## 4. Discussion

This is a prospective cohort analysis reporting the effect of 1 year of therapy with mepolizumab on R5–R20 parameters measured through impulse oscillometry. The significant improvement of R5–R20 values correlates with better PROs. In the era of phenotyping and personalized therapy for patients with asthma, this analysis reveals a novel therapeutic target and endpoint for mepolizumab strategy.

According to the current knowledge, the prevalence of SAD is likely higher in severe asthma and negatively correlates with asthma control and symptom burden [9,23,24]. The ATLANTIS study denoted the correlation between the presence of SAD and the risk of poor asthma control, acute exacerbations and progressive lung function decline, showing how the assessment of SAD is essential for optimizing patient phenotyping [25]. The SASAM study confirmed these data showing that 81% of the severe eosinophilic asthmatics included in this study were affected by SAD.

Farah and co-workers found that an early improvement in SA function, assessed by multiple breath nitrogen washout (MBNW), was associated with better asthma control and markedly contributed to the therapeutic response. However, MBNW is commonly used only in research settings [26]. As for MBNW, various other methods have been suggested to evaluate the periphery of the lungs, despite showing poor inter-test agreement [10,27,28,29]. IOS assessment, being an effort-independent and easy to perform technique, provides a reliable and accurate evaluation of SAD [30,31,32].

The effect of mepolizumab on SAD was previously shown by Antonicelli et al. who reported a significant improvement in a sample of 18 severe asthmatics]. However, they assessed SA through Forced Oscillation Technique (FOT) parameters and did not consider R5–R20 delta as the primary endpoint, as we did in our study. [33] Similarly, Sposato and colleagues observed an improvement in SAD in patients treated with mepolizumab, but the periphery of the lung was measured through an indirect assessment, such as FEF25-75%, in a retrospective observational study [34]. The SASAM study, being prospective, allowed for a planned and comprehensive data collection including both direct and indirect SAD parameters. Abdo et al. studied 20 severe eosinophilic asthmatics treated with an anti-type 2 biological drug (mepolizumab, benralizumab or dupilumab) and demonstrated that the presence of SAD, measured by IOS, was a valuable predictor of clinical response [35]. However, in their study, the net number of subjects undergoing mepolizumab was smaller compared to our sample size for the same primary endpoint.

This prospective observational study highlights the beneficial effect of 1 year of therapy with mepolizumab on small airway disease in severe uncontrolled eosinophilic asthmatics. R5–R20 values, assessed by IOS and considered the primary study endpoint, demonstrated a significant improvement after 12 months of biological treatment. The beneficial effect of mepolizumab was substantially confirmed by the indirect parameters of SAD, specifically, FEF25-75% predicted and TLC% predicted, both showing a concomitant significant improvement after 12 months.

In our study, results from IOS parameters were only partially predicted by consistent indirectly assessed SA parameters. In fact, only FEF25-75% values correlated with R5–R20, and together with TLC% were consistent with R5–R20 at 12 months. This proves the superiority of direct measures in assessing SAD and that R5–R20 should be considered the most valuable tool to evaluate lung periphery.

Interestingly, in our study, the significant correlation between lower R5–R20 values and patient reported outcomes (PROs), measured through the ACT score, also highlights how SAD is able to impact symptom perceptions and clinical control. Indeed, within the first 3 months, the mean ACT score improved by at least 3 points, which is the minimal clinically important difference (MCID) [36]. Additionally, complete clinical control, defined as a mean ACT score of ≥20, was achieved by 12 months. These findings are pivotal for considering SAD as a potential independent endpoint for biological treatment in order to ameliorate severe asthma symptom burden.

In addition, SA histological, radiological and functional abnormalities in severe eosinophilic asthmatic patients have been reported in the literature, despite treatment with maximum dose of ICS/LABA [37,38,39]. This evidence suggests that currently available inhaled therapies might not exert a complete effect on distal bronchi or not adequately achieve optimal peripheral delivery [40,41].

Considering its feasibility in clinical practice, effort independence, cost-effectiveness, and non-invasiveness, IOS is the gold standard for evaluating small airways and represents a relevant added value in our study. Although the SASAM study is a proof-of-concept, it prospectively included a proper sample size to enhance the knowledge in this field.

According to the updated literature, the improvement in SA function might be due to mepolizumab systemic anti-eosinophilic activity which was demonstrated to reach distal bronchi compartments [15,26,29,34,35,39]. To that extent, the SASAM study confirmed that SAD has the potential to be considered a new valuable severe asthma feature and that mepolizumab might be the most effective targeted therapy to treat the distal lung regions.

Our study confirmed all the results of pre- and post-clinical studies [16,17,18] reporting the effect of mepolizumab in severe eosinophilic asthma as early as the first 3 months of observation. In particular, the drug led to a suspension of oral corticosteroids in all the subjects treated with chronic long-term OCS treatment as well as a considerable reduction in asthma exacerbation events, and a significant eosinophil reduction, which is the principal mepolizumab pharmacological effect as an anti-IL5 strategy. The FeNO parameters slightly decreased even if not enough to reach the normality cut-off (<25 ppb) [42], probably due to proper anti-IL5 cellular targeting, which does not specifically affect FeNO production. In terms of functional parameters, only a modest FEV1 increase has been observed in RCTs [30,43]. In this real-life study, patients showed a significant FEV1% improvement after 12 months of therapy, supporting the mepolizumab effect on chronic hyper-eosinophilic inflammation on both small and large airways.

Supporting these findings, Shirai T. and colleagues observed that in those who have achieved clinical remission, IOS parameters improved earlier than spirometry during biological treatment with another anti-eosinophilic strategy such as benralizumab [44]. According to that, SA assessment appears to have even more potential value, being able to detect mepolizumab functional objective benefits not identifiable with standard spirometry. The non-statistically significant improvement in the number of patients affected by SAD over 1 year of treatment, could be partially explained by a dichotomous distinction in groups potentially impacted by a non-unanimously agreed cut-off set at R5–R20 > 0.07 kPa/L/s. In any case, an average amelioration of R5–R20 was paralleled to better asthma outcomes for the whole sample.

One of our study limitations is the observational design, which is exposed to higher observational biases. The SASAM study started during the COVID-19 pandemic which led to delays in properly attending pulmonary function tests and clinical assessments. According to the nature of this study, subjects started biologics following an MDT evaluation and standard clinical practice. Our study does not have a placebo-control arm that would be unethical for this treatment population, because biologic therapy for severe asthma with an eosinophilic phenotype is a well-evidenced and guideline-recommended treatment. Then, the availability of other anti-eosinophilic/T2 strategies impacted the small sample size. Nevertheless, our cohort size is comparable to previous studies where lung function tests, including SAD assessment, were performed to describe a similar population.

This study supports the growing body of evidence highlighting the role of biologics in managing severe asthmatics by adding a focus on the periphery of the lung: an untreated “quiet zone” which is entitled to be more impactful than expected. While the role of SAD as a predictor and marker for response to biological therapy in severe eosinophilic asthma was already recognized before [35], the SASAM study supports the importance of implementing the assessment of the periphery of the lung by performing IOS and the effect of an anti-IL-5 strategy on the small airway. Our data showed that that a more comprehensive effect on small airways is achieved 1 year after the initiation of mepolizumab therapy. This improvement was paralleled with the amelioration in functional parameters, which had not been previously, clearly reported. Considering SAD as an independent variable of response, our findings suggest that targeting this emerging endpoint, which is closely linked to asthma symptoms control and eosinophilic inflammation in the periphery of the lung, might lead to better clinical outcomes. As suggested by our findings, IOS may represent a reliable tool to assess small airways and should be used routinely in patients for severe asthma when choosing the optimal biologic therapy. Whether this effect is attributable to the specific molecule, or the broader class requires further investigation in larger populations, remains to be assessed.

## 5. Conclusions

To the best of our knowledge, the SASAM study first prospectively demonstrates a significant improvement in small airway disease, using a direct measurement of distal lung periphery like Impulse Oscillometry (IOS), undergoing mepolizumab treatment.

Such improvement, detectable after 12 months of mepolizumab treatment, was robustly significantly related to a clinically relevant reduction in asthma symptoms.

Hence, SAD is identified as a highly valuable treatable trait, representing a novel endpoint and a distinct target for optimally managing uncontrolled eosinophilic severe asthmatics and supporting in guiding the choice for the most appropriate biologic therapy when multiple options are available

## Figures and Tables

**Table 1:** Study population baseline characteristics (*n* = 18).

Age (years)		61.1 (±12.0)
BMI		27.1 (±5.10)
Gender	Female	10 (55.5)
	Male	8 (44.5)
Smoking History	Never	13 (72.2)
	Former	4 (22.2)
	Current	1 (5.6)
R5–R20 > 0.07 kPa/L/s (SAD)		15 (83.3)
R5–R20 (kPa/L/s)		0.25 (±0.15)
FEV1L		1.70 (±0.78)
FEV1%pred.		68.0 (±24.1)
FEV1/FVC%		59 (±21.6)
FEF25-75% pred.		31.9 (±23.0)
TLC% pred.		96.4 (±16.2)
RV/TLC%		45.0 (±10.7)
ACT		14.7 (±5.6)
BEC (cell/mm^3^)		261 (220–520)
FeNO (ppb)		47.8 (±36.5)
Medium–high dose ICS/LABAs		18 (100)
LAMA		17 (94)
Patients ≥ 1 AE requiring OCS/previous year		15 (83.3)
mOCS		4 (22)

Data are presented as N (%) for gender, smoking history, atopy, R5–R20 > 0.07 kPa/L, medium–high dose ICS/LABA, LAMA, patients with ≥1 AE requiring OCS/previous year and with continuative OCS therapy; all other data are presented as mean (±SD) or median (IQR) for BEC. mOCS: maintenance OCS.

**Table 2:** Study results (*n* = 16).

	T0	T3	*p*-Value ^T0–T3^	T6	*p*-Value ^T0–T6^	T12	*p*-Value ^T0–T12^
R5–R20 (kPa/L/s)	0.24 (±0.15)	0.25 (±0.21)	0.45	0.19 (±0.18)	0.05	0.18 (±0.18)	0.03 *
SAD—*n* (%)	13 (81)	10 (62)	0.43	10 (62)	0.43	9 (56)	0.13
FEF25-75%pred.	30.4 (±20.6)	38.7 (±25.6)	0.09	47.5 (±34.1)	0.06	41.4 (±28.8)	0.04 *
TLC%pred.	95.6 (±17.1)	100.2 (±12.3)	0.21	101.5 (±17.1)	0.26	104.7 (±9.3)	0.04 *
RV/TLC%	43.9 (±9.55)	39.5 (±10.1)	0.01 *	48.4 (±19.0)	0.26	44.9 (±9.3)	0.12
FEV1%pred.	71.4 (±22.9)	82.3 (±34.3)	0.05	83.1 (±33.4)	0.06	80.8 (±26.7)	0.03 *
ACT	15.1 (5.8)	19.0 (±4.6)	<0.01 *	19.6 (±3.5)	<0.01 *	20.3 (±4.4)	<0.01 *
Patients ≥ 1 AE requiring OCS/previous year	15 (94%)	1 (6.2%)	<0.01 *	2 (12.5%)	<0.01 *	3 (18.7%)	<0.01 *
Patients with mOCS	4 (25%)	0 (0%)	0.10	0 (0%)	0.10	0 (0%)	0.10
BEC (cell/mm^3^)	250 (220–477)	70 (40–130)	<0.01 *	40 (20–111)	<0.01 *	50 (40–90)	<0.01 *
FeNO (ppb)	49.2 (±39.4)	44.5 (±43.6)	0.35	52.8 (±48.5)	0.24	43.1 (±26.4)	0.07

Note: Data are presented as N (%) for SAD, patients with ≥1 AE requiring OCS/previous year; patients with mOCS; all other data are presented as mean (±SD) or median (IQR) for BEC. * Statistically significant. To note, patients with ≥1 AE requiring OCS at T3 and T6 has been adjusted and accordingly compared to 12 months at T0 and T12.

## Data Availability

All data generated or analyzed during this study are included in this article. Further enquiries can be directed to the corresponding author.

## Abbreviations

The following abbreviations are used in this manuscript:

ACT	Asthma Control Test
AE	Acute Exacerbations
BEC	Blood Eosinophil Count
BMI	Body Mass Index
COVID-19	Coronavirus disease-19
FEF25-75%	Forced Expiratory Flow between 25% and 75% of vital capacity;
FeNO	Fractional Exhaled Nitric Oxide
FEV1/FVC	Forced Expiratory Volume in 1 s/Forced Vital Capacity
FEV1	Forced Expiratory Volume in 1 s % of predicted
FVC	Forced Vital Capacity
ICS/LABA	Inhaled Corticosteroids/Long-Acting Beta Agonists
IOS	Impulse Oscillometry
IQR	Interquartile Range
LAMA	Long-acting Muscarinic Agonists
OCS	Oral Corticosteroids
mOCS	Maintenance oral corticosteroids
PROs	Patient Reported Outcomes
R5–R20	Resistance at 5 Hz and 20 Hz
RV/TLC	Residual Volume/Total Lung Capacity
RCT	Randomized Clinical Trial
SA	Small Airways
SAD	Small Airway Disease
SD	Standard Deviation
SEM	Standard Error of the Mean
TLC	Total Lung Capacity
